# Individually Tailored and Culturally Adapted Internet-Based Cognitive Behavioral Therapy for Arabic-Speaking Youths With Mental Health Problems in Sweden: Qualitative Feasibility Study

**DOI:** 10.2196/46253

**Published:** 2023-11-24

**Authors:** Youstina Demetry, Elisabet Wasteson, Tomas Lindegaard, Amjad Abuleil, Anahita Geranmayeh, Gerhard Andersson, Shervin Shahnavaz

**Affiliations:** 1 Centre for Psychiatry Research Department of Clinical Neuroscience Karolinska Institute Stockholm Sweden; 2 Department of Psychology and Social Work Mid Sweden University Östersund Sweden; 3 Department of Behavioural Sciences and Learning Linköping University Linköping Sweden; 4 Competence Team for migration health Region Jämtland Härjedalen Östersund Sweden; 5 Department of Biomedical and Clinical Sciences Linköping University Linköping Sweden

**Keywords:** internet-based interventions, cultural adaptation, Arabic, youth, depression, anxiety, refugees, mental health, Arabic-speaking youth, mental disorder, psychological intervention

## Abstract

**Background:**

Most forcibly displaced refugees in Sweden originate from the Arab Republic of Syria and Iraq. Approximately half of all refugees are aged between 15 and 26 years. This particular group of youths is at a higher risk for developing various mental disorders. However, low use of mental health services across Europe has been reported. Previous research indicates that culturally adapted psychological interventions may be suitable for refugee youths. However, little is known about the feasibility, acceptability, and efficacy of such psychological interventions.

**Objective:**

This study aimed to explore the feasibility, acceptability, and preliminary efficacy of an individually tailored and culturally adapted internet-based cognitive behavioral therapy for Arabic-speaking refugees and immigrant youths in Sweden.

**Methods:**

A total of 17 participants were included to participate in an open trial study of an individually tailored and culturally adapted internet-based cognitive behavioral therapy targeting common mental health problems. To assess the intervention outcome, the Hopkins Symptom Checklist was used. To explore the acceptability of the intervention, in-depth interviews were conducted with 12 participants using thematic analysis. Feasibility was assessed by measuring treatment adherence and by calculating recruitment and retention rates.

**Results:**

The intervention had a high dropout rate and low feasibility. Quantitative analyses of the treatment efficacy were not possible because of the high dropout rate. The qualitative analysis resulted in 3 overarching categories: experiences with SahaUng (the treatment), attitudes toward psychological interventions, and personal factors important for adherence.

**Conclusions:**

The findings from this study indicate that the feasibility and acceptability of the current intervention were low and, based on the qualitative analysis, could be increased by a refinement of recruitment strategies, further simplification of the treatment content, and modifications to the cultural adaptation.

## Introduction

### Background

Approximately 274,000 refugees were hosted by Sweden between the years 2015 and 2020 [1], with more than one-third of the refugees originating from the Arab Republic of Syria and Iraq. Of all refugees from Syria and Iraq, 48% were aged between 15 and 26 years [1]. Previous research has shown that immigration may lead to dramatic changes in an individual’s social, financial, psychological, and cultural status. Immigrants and refugees are a truly heterogeneous group [2-4] with most immigrants and refugees showing high resilience and eagerness to integrate [5-7]. However, for some people, leaving one’s home and moving to a foreign country is associated with the development of various mental health problems [2]. With regard to refugee and immigrant youths specifically, previous research has shown that these groups are at an increased risk of developing mental disorders [8,9]. For example, the risk of developing posttraumatic stress disorder (PTSD) is increased among both unaccompanied and accompanied refugee youths compared with their Swedish counterparts [10]. These findings are also in line with the patterns observed in other European countries [9]. Moreover, the risk of depression and adjustment disorders is higher among refugee youths [8]. The risk of suicide attempts and completed suicides is more pronounced among some refugee subgroups, namely male youths from Afghanistan [11]. Findings from Denmark confirm that suicide attempts and suicide rates are about 5 times higher among refugee youths who arrived in the host country as unaccompanied minors compared with the general population [12]. In Sweden, however, the risk of reattempt and death by suicide are lower among refugees compared with Swedish-born residents [13].

Factors moderating mental health outcomes include the duration of residency in the host country, age, and gender [9,10]. In addition, being a 1.5 generation immigrant or refugee, that is, moving to Sweden before identity formation years, has been documented as a protective factor [10]. In a study from Norway with Syrian refugee youths, postmigration stressors (defined as economical concerns, perceived discrimination, and worrying about migration status) mediated the relationship between potentially traumatic events (ie, experiences from the war) and subjective health-related quality of life [14]. The authors argued that postmigration stressors indirectly enhance the impact of potentially traumatic events and are, in turn, associated with lower subjective health-related quality of life. Longitudinally, elevated health-related problems among refugees who arrive in the Nordic countries before coming of age have been reported in Denmark and Sweden, in particular among men [15].

Despite the abovementioned findings, at the group level, ethnic minority youths’ use of mental health care services is lower compared with their Swedish counterparts [16]. It is worth mentioning that the use of mental health care services among refugee and immigrant youths is dynamic, and there are significant differences among subgroups. For example, it has been reported that this underuse was not present among unaccompanied refugee minors during their first 2 years in Sweden [16]. Unaccompanied refugee minors are usually appointed a contact person and use of services can be initiated by the contact person rather than by the youths. Disparities in the use of mental health care services between unaccompanied refugee minors and their Swedish counterparts increased after the initial 2 years upon arrival. The underuse of mental health care services among refugee and immigrant youths has also been observed in other European countries [17]. The combination of a higher risk for mental health problems and underuse of mental health services raises concerns for this susceptible group.

Cultural differences, social factors, financial issues, and language have been identified as explanations for the low use of health care services among refugee youths [18,19]. Barriers such as mistrust of services and concerns regarding privacy have been reported [19]. In addition, lack of knowledge of mental health problems and the perception of psychological services as being ineffective are also brought up as barriers. Moreover, language issues include poor interpretation quality and lack of information on mental health problems in different languages. Stigma and beliefs about mental health problems are brought along from the country of origin and are reported as barriers against the use of mental health services [19]. It has been suggested that even well-integrated individuals can “swing...towards cultural influences that hold traditional beliefs” [20] when experiencing stressful situations. Financial barriers including logistics related to getting to the health care centers have been suggested [21]. Finally, some immigrant groups such as adult asylum seekers have restricted access to health care services according to the Swedish law. It is argued that internet-based interventions can overcome these barriers [22]. For instance, internet-based interventions facilitate access to services in the respective language of the patient [23]. Moreover, internet-based interventions allow for access to services anonymously, which may be preferable for our target population owing to stigmatization [24]. We believe that internet-based cognitive behavioral therapy (iCBT) may be acceptable for our target population. In addition, the cultural adaptation of evidence-based interventions may also address a number of the abovementioned barriers.

### What is Cultural Adaptation?

Cultural adaptation is defined as modifying interventions to fit clients’ cultural beliefs, values, language, context, meanings, and behaviors [25]. Several frameworks and guidelines for cultural adaptation have been proposed [25,26]. For the purpose of this paper, the focus is on the guidelines for cultural equivalence as described by Helms [26].

In this framework, 3 aspects of cultural equivalence are identified when culturally adapting interventions: functional, conceptual, and linguistic equivalence [26]. Functional equivalence refers to adapting to the culture of interest’s interpretation of and reactions to various behaviors. Conceptual equivalence is defined as reflecting various cultural constructs. To give an example, attitudes toward seeking psychological help is such a cultural-specific construct that needs to be considered in the adaptation process. Linguistic equivalence refers to the extent to which the language used in the intervention bears meanings relevant to the specific cultural group.

### Why is Cultural Adaptation Necessary?

High attrition rates have been reported among youths who belong to ethnic minorities in intervention studies [20]. Cultural aspects (ie, meanings, values, norms, and beliefs) influence the presentation of explanatory models regarding the emergence and maintenance of symptoms [27] and also have implications for treatment. Not taking these factors into account when providing an intervention may result in disengagement and eventually attrition [20].

There is also a gap in the literature on effective interventions for immigrants and refugees. The few meta-analyses that have been conducted to explore the efficacy of culturally adapted interventions for adults revealed moderate effect sizes in favor of adapted interventions when compared with nonadapted interventions [28,29]. However, the efficacy of culturally adapted interventions for youths is inconclusive [30,31], and best practices for cultural adaptation of interventions for youths are yet to be determined [32].

In light of the abovementioned aspects, it is important to further investigate the cultural adaptation of interventions targeting ethnic minority youths. As suggested by Rathod and Kingdon [33], the pragmatic nature of CBT and its long history of evidence makes it a fertile soil for cultural adaptation.

### CBT and the Internet

CBT is a recommended treatment for common mental health problems such as depression and anxiety in children, youths, and adults [21]. For children and youths, a recent meta-analysis showed that iCBT resulted in moderate effects for psychiatric and somatic conditions in comparison to waitlist control conditions [34]. The acceptability, feasibility, and efficacy of iCBT for refugees and migrants have been investigated previously, but mainly for adults [35,36]. Patterns of effectiveness and adherence of culturally adapted internet-based interventions are similar to those of internet-based interventions targeting the general population. These interventions are effective but have low adherence. One way to maneuver this is by developing tailored interventions that target various mental health problems. This may be particularly beneficial for refugees and immigrants where comorbidity is expected. A limited number of studies have investigated tailored iCBT [37]. With that said, the ones that have found it to be effective for anxiety and depression for various populations [38-41]. To our knowledge, only one previous study has explored an internet-based intervention for refugee youths and young adults, in this case targeting Farsi and Dari-speaking youths [31].

### Aims

This study aimed to explore the feasibility, acceptability, and preliminary efficacy of a culturally adapted iCBT intervention for Arabic-speaking youths in Sweden with symptoms of common mental disorders.

## Methods

### Study Design and Recruitment

This study used a pre-post open study design. Furthermore, in-depth interviews with a group of Arabic-speaking youths who were not in treatment were conducted. Participants were recruited over a 3-month period, ranging from November 1, 2021, to January 31, 2022. Facebook paid advertisements targeting Arabic-speaking youths aged between 18 and 26 years of both genders were used to promote registration to the study. It was not possible to promote paid advertisements for participants below the age of 18 years owing to Facebook’s advertising policy. In addition, information regarding the study was distributed through various other Facebook pages for psychologists, researchers, and various groups for Arabic-speaking newly arrived immigrants and refugees in Sweden. The study was promoted through a collaboration with influencers and social entrepreneurs who were followed by the target population. Finally, emails were sent to organizations working with the target population. Participants for the in-depth interviews (individuals who belonged to the same language group but did not participate in the treatment study) were recruited through (1) a research and training center in Egypt and (2) posts on a social media platform.

### Procedure

Participants gave their consent to participate on the study web page. Once participants provided informed consent, an email was sent with a link to fill out a screening measure, pretreatment assessment (see the subsection *Materials*), and demographic information. Participants who met the initial screening criteria participated in a 30-minute clinical interview before inclusion to the study. This clinical interview aimed to assess the potential presence of severe mental health problems such as psychosis and severe depression. In addition, the interviews assessed suicidality and substance dependency. Research team members held weekly meetings to assess the eligibility of the newly interviewed participants. Once included, a member of the research team telephoned the included participant to start up participation in the intervention. Participants were also assigned a language-matched therapist for guidance and weekly follow-ups. The weekly follow-ups comprised telephone calls ranging from 5 to 25 minutes and focused on discussing the respective week’s exercises, potential challenges, and choosing the next module. Upon completing the assigned modules (which varied depending on the participant’s problem areas), participants received a new link to fill out posttreatment measures. Finally, an in-depth semistructured interview was conducted with participants regarding the acceptability and cultural relevance of the intervention.

In addition, 3 nontreatment participants were interviewed to assess the acceptability of the treatment material. These 3 participants were provided with 3 treatment modules. They were given 2 weeks to go through the material after which they were invited to participate in a semistructured interview to assess its acceptability and cultural relevance of the material. The nontreatment participants were interviewed before starting the recruitment of participants to the intervention (Multimedia Appendix 1; [31]).

### Participants

**Figure 1:**
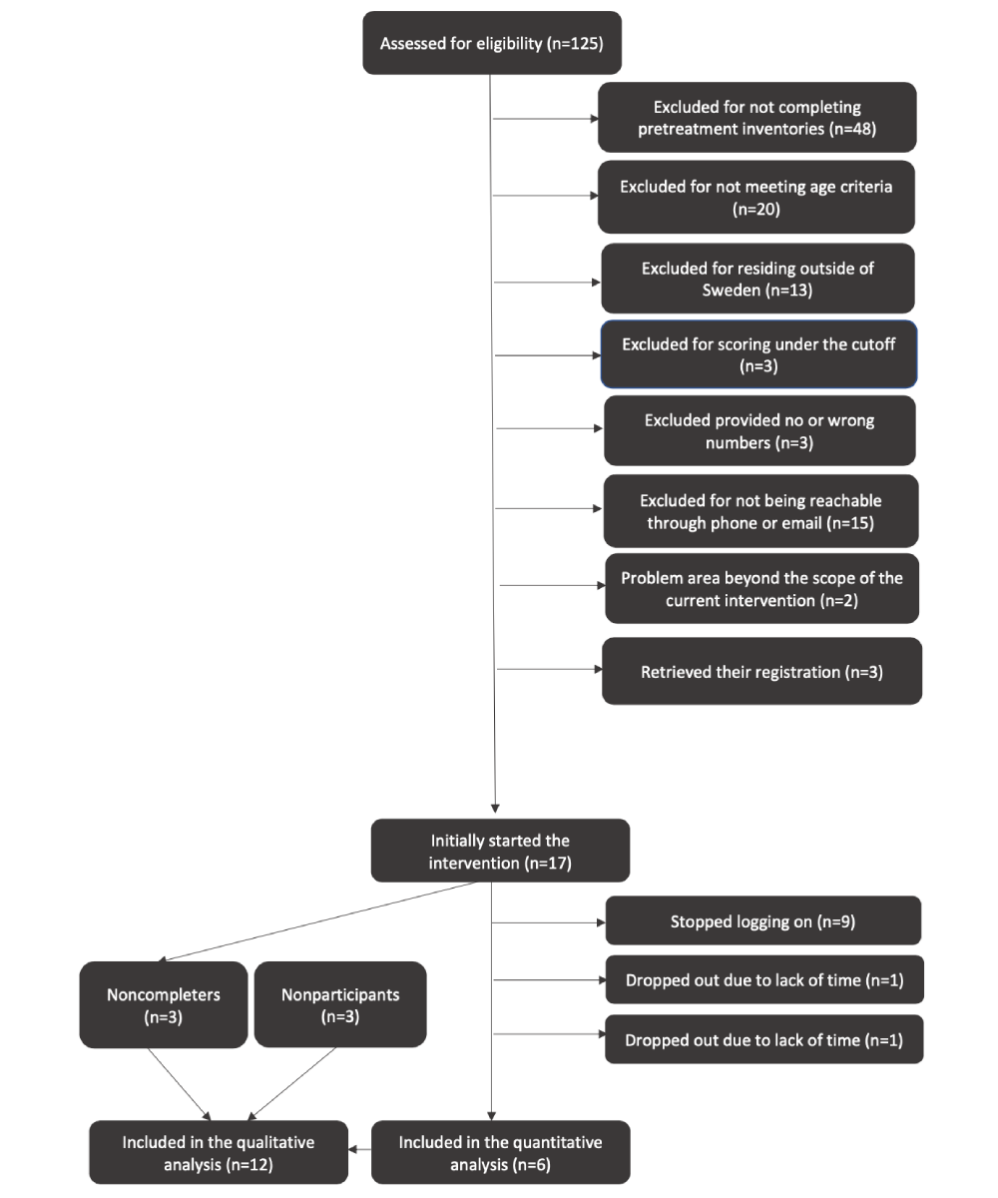
Participants flowchart.

For this study, a power analysis was not conducted because of the nature of this study, that is, focus on feasibility. As shown in Figure 1, a total of 125 individuals registered on the study platform. Of these, 107 (84.8%) were excluded for the following reasons: (1) did not complete the pretreatment measures (n=48, 45.3%); (2) were older than 26 years of age (n=20, 18.9%); (3) did not reside in Sweden (n=13, 12.3%); (4) scored under the cutoff of the screening measure (n=3, 2.8%); (5) withdrew their registration (n=3, 2.8%); (6) provided no or wrong phone numbers (n=3, 2.8%); (7) were not reachable through phone or email after registration (n=15, 14.1%); and (8) reported problem areas that were beyond the scope of the current intervention; namely problems related to sexuality or sexual dysfunctions (n=2, 1.9%). A total of 17 participants initially started the intervention. To deepen our understanding of the extent to which the intervention was acceptable and culturally relevant, interviews were conducted with nontreatment participants and included in the qualitative analysis. Nontreatment participants comprised 3 individuals varying in age, language proficiency, and knowledge of psychological problems, all with an Arabic cultural background and aged between 15 and 26 years.

### Ethical Considerations

This study is a part of a larger project, namely “SAHA-project,” which has been ethically reviewed and approved by the Swedish Ethical Review Authority (2020-03290). The participants provided informed consent through the intervention website before registering to the intervention. The participants did not receive any compensation for their participation in the intervention. To ensure privacy, data were pseudonymized. To ensure confidentiality protection, only research team members have access to the intervention platform and log in is only possible through 2-step authentication.

### SahaUng’s Intervention, Cultural Adaptation, and Treatment Platform

Initially, the Saha (صحة meaning *health* in Arabic) intervention was developed for Arabic-speaking immigrant and refugee adults [42]. The Saha intervention for adults consists of the following 9 modules: introduction, depression, anxiety, insomnia, emotion regulation, posttraumatic stress, stress, rumination, and termination. A 10th module focusing on loss and grief was added to the second version of the Saha intervention, SahaUng, which is an adapted version of the original Saha intervention targeting refugee youths experiencing common mild to moderate mental health problems. This, in addition to the Arabic version described here, has been translated and adapted into Dari or Farsi [31]. SahaUng (the youth-adapted version) is a tailored intervention in which the introduction module and maintenance module are mandatory, and the other modules are assigned based on the needs of the participants. Participants were required to complete at least 3 modules. The choice of modules was based on various factors. These include the answers provided in PSYCHLOPS, the scores of Hopkins Symptom Checklist (HSCL)-5, the presence of symptoms of either subclinical PTSD or subclinical complicated grief (see *Measures* section). Furthermore, together with the therapist, the participants ranked the priority of the different problem areas. This was done to determine the order and range of modules needed for each participant.

For SahaUng, a top-down cultural and language adaptation of the intervention was used based on the guidelines of cultural equivalence and the frameworks implemented in a previous culturally adapted internet intervention [26,43]. As part of the cultural adaptation, the participants were matched with an Arabic-speaking therapist. To further accommodate for the needs of youths, the language used was simplified and psychoeducational videos were added to each module. Such accommodations were based on the results from the Farsi/Dari iCBT pilot study for young refugees [31] and the semistructured interviews conducted with the 3 nontreatment participants. Multimedia Appendix 2 provides the details on the cultural adaptation [31].

The SahaUng intervention was made available on Iterapi [44], a web-based platform for psychological interventions developed at Linköping University, Sweden [45].

### Measures

#### Primary Outcome Measure

The HSCL-25 was used as the primary outcome measure [46]. The 25 items in the HSCL-25 measure anxiety and depressive symptoms. The first 10 items focus on anxiety symptoms and are followed by 15 items for depressive symptoms. The items are rated on a 4-point Likert scale. On the basis of the previous studies, a cutoff of 1.75 was used as one of the inclusion criteria. The Arabic version of the HSCL-25 has been validated and used previously in various studies with Arabic-speaking participants [47].

#### Secondary Outcome Measures

The PSYCHLOPS ask participants to describe the problem that currently affects them the most [48]. The patient then responds to 4 questions regarding how affected they are by the problem, their overall well-being, and how affected they are by a secondary problem. These were scored on a 5-point Likert scale ranging from 0 to 4. Preliminary research in the primary care setting suggests that PSYCHLOPS is a sensitive and reliable measure of change following psychological treatment [48]. The Arabic version of PSYCHLOPS has been used in previous studies [49].

To assess sleep quality, we used the Insomnia Severity Index (ISI) [50]. The ISI is a 7-item questionnaire using a 5-point Likert scale. Higher scores indicate a higher degree of severity regarding insomnia. The ISI has been validated in Arabic [51].

Participants were asked whether they had lost an important person. Those who indicated the loss of a dear one completed the Prolonged Grief Disorder-13 (PG-13) [52]. The PG-13 consists of 13 items rated on a 5-point Likert scale. The Arabic version of PG-13 was used previously in a Saudi Arabian context [53].

In case of having experienced traumatic events, participants filled out the Posttraumatic Stress Disorder Checklist (PCL-5) [54]. The PCL-5 consists of 20 items and assesses symptoms of PTSD according to the Diagnostic and Statistical Manual of Mental Disorders, Fifth Edition. Responses were recorded on a 5-point Likert scale. The PCL-5 has been validated in Arabic-speaking participants [55].

Finally, to measure the overall satisfaction with the intervention, the Client Satisfaction Questionnaire-3 (CSQ-3) [56] was used after the treatment. The questionnaire consists of 3 items and responses are provided on a 4-point Likert scale. A higher score indicates a higher level of satisfaction with the intervention. CSQ-3 has been validated and used in previous studies with Arabic-speaking participants [57].

#### Feasibility

##### Overview

Feasibility was assessed by collecting data on recruitment and retention rates. Retention rates have been used as a measure of feasibility in previous studies [58]. Facebook advertisements result rates were calculated using a simple equation of website clicks divided by impressions. Furthermore, the time required to recruit the desired sample size was used as one of the measures of feasibility. In addition, adherence rates were included as a measure of feasibility.

##### Pretreatment Clinical Assessment Interview

A semistructured interview comprising 14 questions was developed by research team members to assess (1) current problem areas, (2) psychosis or severe psychological disorder, (3) suicidality, (4) current medication, (5) substance use, (6) motivation, and (7) availability of external support.

#### Acceptability

The acceptability of the intervention was measured using 2 different semistructured interviews.

#### Posttreatment Semistructured Interview for Participants

A semistructured interview was developed to explore acceptability and cultural relevance of the intervention. The interview consisted of 29 questions focusing on (1) background information, (2) experiences with the platform, (3) experiences regarding contact with therapist, (4) cultural perceptions of psychological illnesses, (5) acceptability of the intervention, and (6) cultural relevance of the intervention. With regard to acceptability, questions included in the interview guide were based on the acceptability theoretical framework developed by Sekhon et al [59]. The framework consists of the following 7 domains: affective attitude, burden, perceived effectiveness, ethicality, intervention coherence, opportunity costs, and self-efficacy. In terms of cultural relevance, 4 questions were adapted from the cultural relevance questionnaire developed by Salamanca et al [43]. Multimedia Appendix 1 provides the complete interview guide [31].

#### Semistructured Interview for Nontreatment Participants

Another semistructured interview was developed to explore acceptability and cultural relevance before starting the intervention. The interview guide consisted of 26 questions. The questions focused on (1) demographics, (2) technical difficulties with the platform, (3) acceptability, and (4) cultural adaptation and relevance. Multimedia Appendix 1 provides the complete interview guide [31].

### Analyses

#### Quantitative Analysis

Statistical analyses of treatment efficacy were initially planned. However, owing to the high dropout rate, it was not possible to conduct any statistical analyses with regard to treatment effects. Therefore, only descriptive statistics are presented. Descriptive statistics were based on data collected from completers, which in this study were defined as participants who took part in at least 3 modules and completed both pre and posttreatment questionnaires.

#### Qualitative Analysis

The interviews were analyzed by the first author using thematic analysis [60]. Familiarization with the interviews took place by transcribing, translating, and reading the interviews several times and highlighting relevant phrases. The initial coding scheme was based on 2 interviews. This was revised, refined, and changed over time as more interviews were coded. To explore reflexivity, the second author independently coded relevant phrases in 2 of the interviews. The highlights of the first and second authors were cross-referenced and reflected upon. Disagreements were discussed until consensus was reached.

The initial coding was followed by grouping the codes and creating subthemes, themes, and overarching categories. The development of themes was descriptive in nature; however, it is important to acknowledge that the analysis is always influenced by the researchers’ own theoretical and epistemological orientation and background, and that this is considered an inherent part of the analysis process in thematic analysis [61]. The initial coding was conducted by the first (YD) and second (EW) authors. Finally, the fourth author (AG) and the last author (SS) reviewed the coding scheme and interviews. Once they provided their feedback, a revalidation and refinement of the coding scheme was conducted by the first author (YD). A final revalidation and refinement of the coding scheme was conducted by all the authors in conjunction with the write-up of this paper.

## Results

### Quantitative Results

#### Feasibility and Clients’ Satisfaction

Over a period of 3 months, 4 paid Facebook or Instagram advertisements were created. A total of 38,022 impressions and 899 clicks (to the website) were obtained. Thus, the result rate of the Facebook advertisements was 2.1%. A total of 125 (13.9%) potential participants registered for the intervention. Of these, only 17 (13.6%) fulfilled the inclusion criteria. Of the 17 participants, 6 (35%) completed the intervention.

Among the completers, an average of 4.6 (SD 0.82) modules were completed, whereas noncompleters completed 1.5 (SD 1) modules on average. On average, completers completed 71.75% of the assigned exercises, whereas noncompleters completed an average of 27.56% of the assigned exercises. Similarly, the average number of logins was higher among completers when compared with noncompleters (a mean of 21.3 and 4.3 times, respectively). With regard to client satisfaction, participants scored a mean of 10 points (SD 0.894) on the CSQ-3 self-report questionnaire. Table 1 illustrates the modules in which the completers participated.

**Table 1:** Modules completers took part in (N=6).

Module	Percentage of participants who took part in the module, n (%)
Introduction	6 (100)
Depression	5 (83.3)
Anxiety	2 (33.3)
Sleeping problems	1 (16.7)
Stress	2 (33.3)
Rumination	5 (83.3)
Emotion regulation	0
Traumatic events	2 (33.3)
Grief and loss	1 (16.7)
Maintenance	6 (100)

#### Descriptive Statistics

Table 2 presents the demographic characteristics of the participants. In terms of dropout, one participant expressed a wish to drop out because of a lack of time. Another participant wished to drop out because of the feeling that the program was too generic and did not address their specific problem area. Furthermore, 9 participants passively dropped out because of inactivity. Table 3 illustrates the descriptive statistics before and after the treatment.

The intended intervention duration of 10 weeks was not followed for all the participants. Overall, 3 participants participated for 13 weeks because either the therapist or the participant fell ill during the intervention period. On average, the treatment lasted for 10 weeks, ranging from 5 to 13 weeks. Participants completed the posttreatment measurements upon completing the number of modules assigned to them.

**Table 2:** Demographics of treatment participants.

			Completers (N=6)			Noncompleters (N=11)	
			n (%)		Mean (SD)	n (%)	Mean (SD)
**Country of origin**							
	Palestine	1 (16.7)		—^a^		4 (36.4)	—
	Syria	5 (83.3)		—		5 (45.5)	—
	Iraq	0 (0)		—		1 (9.1)	
	Lebanon	0 (0)		—		1 (9.1)	
**Education**							
	Primary education	0 (0)		—		3 (27.3)	
	High school	4 (66.7)		—		7 (63.6)	—
	University	2 (33.3)		—		1 (9.1)	—
**Gender**							
	Male	0 (0)		—		3 (27.3)	—
	Female	6 (100)		—		8 (72.7)	—
**Prior therapy**							
	Yes	1 (16.7)		—		2 (18.2)	—
	No	5 (83.3)		—		9 (81.8)	—
**Marital status**							
	Married	2 (33.3)		—		4 (36.4)	—
	Single	4 (66.7)		—		7 (63.6)	—
Age			—		22.6 (2.5)	—	22.0 (2.9)
Time in Sweden			—		5.3 (1.51)	—	5.9 (1.64)

^a^Not available.

**Table 3:** Descriptive statistics before and after treatment.

	Before treatment			After treatment	
	n	Mean (SD)	n		Mean (SD)
HSCL-25^a^	17	2.94 (0.50)	6		2.01 (0.52)
PSYCHLOPS	17	17.12 (3.08)	6		9.66 (5.82)
ISI^b^	17	16.65 (4.65)	6		13.83 (7.52)
PCL-5^c^	12	49.42 (18.78)	3		31 (26.91)
PG-13^d^	9	31.67 (8.49)	2		29.5 (7.78)

^a^HSCL-25: Hopkins Symptom Checklist-25.

^b^ISI: Insomnia Severity Index.

^c^PCL-5: Posttraumatic Stress Disorder Checklist.

^d^PG-13: Prolonged Grief Disorder-13.

### Acceptability: Qualitative Results

The qualitative analysis was based on 12 interviews. These interviews were conducted with 6 completers, 3 noncompleters, and 3 nontreatment participants. The research team decided to include results from the 3 nontreatment participants to enrich our understanding of the acceptability of the intervention.

Three overarching themes emerged: *experiences regarding SahaUng, attitudes toward psychological interventions,* and *factors important to seeking and adhering to therapy* (Textbox 1)*.* Under experiences regarding SahaUng, 2 themes emerged, namely, *“exceeding all expectations”* and *“room for improvements.”* Under *attitudes toward psychological interventions*, one theme emerged, *cultural attitudes toward psychological interventions.* Finally, under *factors important to seeking and adhering to therapy*, 2 themes emerged *motivations* and *barriers.* Ten subthemes emerged from the mentioned 5 themes. Notably, the names used in the quotes below are pseudonyms.

**Overarching themes and subthemes emerging from the semistructured interviews.**

**Experiences regarding SahaUng**

- Exceeding all expectations:
  Acceptability
  Beneficial components
- Room for improvements:
  Issues with content or format
  Perceived effective intervention strategies

**Attitudes toward psychological interventions**

- Cultural adaptation toward psychological interventions:
  Turning a blind eye
  Mental illness as a flaw

**Personal factors important to adherence**

- Motivations:
  Psychological status
  Intervention-related aspects
- Barriers:
  External barriers
  Internal barriers

#### Experiences Regarding SahaUng

Under the overarching theme “experiences regarding SahaUng,” 2 themes emerged. The first theme was named “exceeding all expectations.” The second theme was named “room for improvements” to reflect negative opinions as well as subjective opinions regarding effective intervention strategies.

#### Exceeding All Expectations

The name of this theme is a citation from one of the participants and was used to reflect the positive opinions regarding the intervention. To avoid misunderstanding, it is necessary to clarify that as much as this quote reflects the perceived acceptability of the intervention, it also reflects the low expectation among participants before the intervention. Under this theme, 2 subthemes emerged: namely, *acceptability of the intervention* and *beneficial components*. The subtheme named “acceptability” reflects views on how easy and likeable the participants found the intervention to be. Moreover, the subtheme named “beneficial components” reflects areas of the intervention which the participants perceived as most beneficial. All completers, one noncompleter, and one nontreatment participant expressed that the platform was easy to navigate and that the intervention’s text was easy to understand. Many participants expressed an overall satisfaction with the intervention, as exemplified by the following quotation:

It is easy to understand, the knowledge quizzes were good, the poems were good and exciting. I can’t think of something negative. As mentioned, I don’t want to finish the program. I want to start it all over again…I tried to put my life together before the program, but it is something else when someone is following up.

Female, 24 years old, completer

One interesting aspect that emerged in the above quote is the participant’s wish to participate all over again in the intervention. From the preceding words, one may assume that the participant appreciated having someone who follows up.

With regard to beneficial components, the intervention was seen to prevent the development of more serious mental illnesses as illustrated by the following quote:

It [the intervention] gives immunity. You receive information that helps you track your illness. [it is like] getting the necessary keys to not become depressed for real…and not relapse.

Female, 19 years old, completer

Participants expressed that it was beneficial to learn to put labels on their problems and appreciated the sense of collectiveness in not being alone in the problems attained through the cases used in the intervention:

You learn to name your problems. Through the cases, you learn that you are not alone in the suffering…that others went through it and now they are feeling better.

Female, 24 years old, completer

A key beneficial component as expressed by many participants related to being paired with a therapist. A well-known Arabic proverb states that “paradise without people is not worth going to*.”* This proverb is a living practice among individuals from Arabic-speaking cultures, and the participants in the current intervention are no exception. This is exemplified by the following quotation:

…the reoccurring calls from the therapists and their attempts of helping me gave me positive feelings. It implied that you truly care to help me get out of the current state in which I live in.

Male, 18 years old, noncompleter

#### Room for Improvement

There are 2 subthemes under this category: issues with content/format and perceived effective intervention strategies.

- Issues with content or format: Technical issues mentioned by several participants included the low quality of videos and having to manually save their answers to avoid losing the data. Furthermore, some participants reported that clicks led them to the wrong tab. The interface of the platform was perceived as unmodern. Although older participants indicated that they did not mind the unmodern interface, they expressed that this may affect the engagement of younger participants.

Among the noncompleters and nontreatment participants, texts were perceived as too long and complicated. Related to this, it seemed that many participants experienced a need for greater support from the therapists. “A mountain on the back*”* is a common way of expressing elevated levels of responsibilities or at times mental burdens in Arabic. Participants expressed that short follow-up calls with a therapist were associated with a feeling of “carrying the responsibility,” which was unfavorable, as illustrated by the following quote:

The calls were too short…I felt like that the responsibility is on me. I would have liked less responsibility, especially at the beginning. Then you could’ve gradually increased the responsibility.

Female, 19 years old, completer

- Perceived effective intervention strategies: Completers have highlighted the need to increase mental health awareness. In addition, some mentioned social media platforms specifically as a means for increasing awareness:

Some have already broken the taboo, but others haven’t yet. But I have to say that it is through increasing awareness…we need to increase trust in psychiatry. If you need to seek help, you need to believe that the therapist can actually help you. You need to believe that you will get results.

Female, 19 years old, completer

Furthermore, participants suggested video calls as an effective intervention strategy. A number of completers also suggested that many refugee youths are in need of a role model and a companion to comply with interventions:

What happens, from a personal point of view, is that youth need to feel that someone cares. You need to show that you care and that you want to help them feel better…Convey that you are there and that you want to help and that they are not alone in whatever they are going through.

Female, 21 years old, completer

Replacing labels associated with mental illnesses with less stigmatizing phrases was also expressed as an essential variable to motivate Arabic-speaking youths to seek help:

You must express yourself cautiously. Don’t say psychiatry. Don’t say psychiatrist or psychologist. We have a problem with the question ‘do you need a psychologist?’. You can replace it with something more lenient. For instance, one may ask ‘is there something that’s upsetting you?’…Because sometimes, it feels like we are magnifying the problem when one says that one needs a psychologist.

Female, 24 years old, completers

#### Attitudes Toward Psychological Interventions: Cultural Attitudes Regarding Psychological Interventions

There are 2 subthemes: turning a blind eye and mental illness as a flaw.

- Turning a blind eye: Participants described that a lack of self-insight and awareness regarding the need for psychological interventions hinders help-seeking. In addition, many people with an Arabic cultural background live in denial according to participants, as illustrated by the quote below:

They deny. They never admit…It is prohibited. Not because it [mental illnesses] doesn’t exist, but they never admit that mental illnesses exist…they explain it with religion…that it has to do with not being close to God.

Female, 19 years old, completer

As reported by the participant in the above quote, religious explanatory models of symptoms can also constitute a barrier to seeking psychological treatment and might make the individual more inclined to turn to traditional healing practices. In addition, many participants believed that self-insight is an essential first step in the right direction toward seeking help for psychological problems, as illustrated by the following quote:

Insight is half-way to well-being…For some people, they don’t have the ability to evaluate the situation and understand that this is a disorder.

Female, 24 years old, completer

- Mental illness as a flaw: A recurring idea in most interviews was that suffering mentally is a flaw. Flaw is a translation of the Arabic word “*eib,*” where a precise English equivalent is difficult to find. As Salarvan [62] (during a discussion by experts in the field) described it, “*eib”* falls somewhere between wrongful and sinful:

It is a huge challenge to tell someone that you are in therapy. You automatically get a lower status when you tell that you are seeing a psychologist. [it is] a flaw (eib)...they say she is crazy. How will she raise children!

Female, 24 years old, completer

It is difficult to receive social support when one expresses that one has a psychological problem. This in turn affects help-seeking behavior. There appears to be a stigma regarding being mentally ill, sharing with others about one’s own mental sufferings and revealing that one is receiving help from mental health care services, as illustrated by the following quote:

In the Arabic culture, there are no guidelines regarding psychological support and how to seek help. When someone suffering from somatic problems, they are motivated [by others] to go to a physician. You don’t get the same support when you are suffering from psychological problems...it was very rare to hear about someone who goes to a psychologist when we lived in Syria. It is seen as embarrassing.

Female, 24 years old, completer

#### Factors Important to Seeking and Adhering to Therapy

Two themes emerged under this overarching theme: motivation and barriers. Two subthemes emerged under “motivation”: psychological status and intervention-related factors.

#### Psychological Status

Both completers and noncompleters described feeling unwell as a motivation to seek help. Interestingly, a number of participants indicated that they sought help for somatic symptoms before they realized that their suffering was better explained as psychological problems. This is illustrated by the following quote:

I had a problem with my stomach...it was very embarrassing. I contacted a physician through KRY [an online health-tech app]. He asked me a number of questions. At last, he drew the conclusion that it is a psychological problem. He recommended that I seek psychological help. A psychologist or something similar. He said that I probably think too much. I said okay but I need an Arabic-speaking psychologist.

Female, 24 years old, completer

#### Intervention-Related Factors

Several completers and noncompleters were motivated to seek help when they found that the intervention was given online and in their mother tongue:

I really liked that such a program exists. It was online and for Arabic-speaking youth in Arabic. I felt that this will be good and that I will be able to talk to someone who would understand me fully. This person [the therapist] will, surely, have knowledge of my background.

Female, 26 years old, completer

As reported by the participant in the above quote, the prospect of being matched with a therapist with a similar cultural or language background increased the motivation for participating in the intervention.

#### Barriers

Two subthemes emerged under “barriers”: external barriers and internal barriers. External barriers are defined as those that are difficult for the individual to control. In contrast, internal barriers are related to one’s own perceived abilities and level of efficacy.

- External barriers: Being busy with daily problems such as job seeking and missing one’s family are examples of barriers that impact adherence to the intervention:

I’m under stress at work. My family is far away from me and live in Syria. They live at risk and are in danger. I’m currently dealing with a financial crisis…my employment contract will end and I’m looking for a new job…

Male, 18 years old, noncompleter

Some participants also expressed that individuals in their environment reacted negatively to them when they tried to implement the exercises related to the intervention, as illustrated in the following quote:

It has nothing to do with the program. Other people just think it is weird when I carry out the exercises.

Female, 26 years old, completer

Mindfulness exercises and behavioral activation were some of the exercises that the participants mentioned in this context.

- Internal barriers: Lack of self-discipline, routine, and concentration were described as barriers to adherence at times, as illustrated by the following quote:

I have trouble with focusing on one thing at the time and I don’t have [self]-discipline. It has to do with me and not the program.

Female, 26 years old, completer

Finally, and somewhat paradoxically, experiencing psychological problems was seen as a barrier, as illustrated by the following quote:

The problem is that it is impossible to sit at night and read, with the aim of getting psychologically better, when the person is psychologically ill, living under stress to the point of exploding.

Male, 18 years old, noncompleter

As reported by the participant in the above quote, the psychological symptoms experienced by this participant seemed to interfere with his ability to concentrate and engage with reading the materials in the treatment program, thereby limiting his ability to make use of the treatment in its current format, with similar difficulties being mentioned by several others of the noncompleters.

## Discussion

### Principal Findings

The aim of this study was to explore the preliminary feasibility, acceptability, and efficacy of an iCBT intervention for Arabic-speaking youths with common mental illnesses in Sweden. This study had low feasibility. Several aspects, including an inability in reaching the targeted population, may have contributed to the low feasibility. No statistical analyses with regard to treatment effects were conducted because of the high dropout rate and small sample size. Previous studies targeting nonrefugee youths have found that iCBT is effective in reducing symptoms among adolescents [34]. It is essential that future studies of iCBT adapted for migrant and refugee youths modify the recruitment strategies and address the high attrition levels to facilitate statistical analyses and draw conclusions regarding the efficacy of such interventions.

The qualitative analysis resulted in 3 overarching themes: namely, *experiences regarding SahaUng*, *attitudes toward psychological interventions,* and *personal factors important to adherence*. Briefly, the acceptability of the intervention was mixed. Participants expressed that the intervention was helpful and that the therapist’s guidance was favorable. However, the length of the texts was considered a burden by some participants. This seemed to be particularly true for young participants and noncompleters. Furthermore, the low quality of videos was considered unfavorable. Generally, cultural factors such as attitudes toward psychological interventions, awareness regarding mental illnesses, and fear of stigmatization seemed to affect both help-seeking behavior and completion of the intervention. Previous research has reported cultural factors such as lack of mental health awareness among Syrian adults [27] and mistrust in the system among refugee youths [19] constituting barriers to treatment seeking and engagement. Our findings reflect similar cultural barriers among youths. To a large extent, the motivational factors and barriers that emerged in this study also emerged in our previous study on Farsi or Dari-speaking youths [31]. Concerning SahaUng, the participants found the content to be beneficial and easy to understand and the platform easy to navigate. Personal factors seemed to play a role in adherence. Generally, feeling unwell was described as a motivation to seek and at times adhere to the intervention, but it could also constitute a barrier to some participants as it made it more difficult to engage with the intervention. Personal barriers included a lack of concentration, motivation, and routine.

With regard to feasibility, this study had recruitment issues. A low budget may have affected the reach of the advertisements. Moreover, Facebook policy did not allow for targeting participants aged between 15 and 18 years. Advertisements were created by nonexperts in this manner (ie, research team members). All of this may have played a role in only 28% of the registered participants fulfilling the initial inclusion criteria. With that, the intervention had a high dropout rate, with only 35% of the participants completing the intervention. Nonadherence to internet interventions is not a novel finding [63]. In a summary of the effectiveness of iCBT for children and adolescents, the authors highlight that low compliance and engagement is a rule rather than an exception for our target population [64]. For example, several of the mentioned interventions on iCBT for depression among adolescents have a completion rate of approximately 25%. The authors resonate with the probable need of more therapist guidance in interventions targeting adolescents. Other feasibility studies on youths have found similar trends in adherence [65]. Low adherence was found in culturally adapted internet-based interventions targeting adults [36,66] as well as youths [31]. It has been suggested that culturally adapted internet interventions are missing additional components to improve user experience [36]. Such findings are in line with the experiences of our participants who have commented on the quality and lack of “modernization” of aspects of the interface. However, previous studies have failed to find a relationship between user interface and level of attrition or engagement [66,67]. Therefore, one needs to consider other factors that may explain the lack of adherence and the high dropout rate in the current intervention. Limited concentration, routines, and level of motivation were identified as barriers to adherence by the participants. This mirrors the findings from our previous study on internet-based CBT for youths and young adults with Farsi/Dari as the mother tongue [31].

### Strengths

This study has several strengths. Findings from the qualitative data enrich our understanding of potential ways to break the barriers of help-seeking among this susceptible group of youths. Furthermore, the participants’ behaviors (in terms of the number of log-ins, choice of modules, etc) will guide our future work and may be helpful for future similar studies. To our knowledge, this is the first study to explore culturally adapted internet-based interventions for Arabic-speaking refugee youths. Through our qualitative work, we have gained a wider understanding of appropriate cultural modifications for internet-based interventions.

### Limitations

The limitations of the study include the small sample size and high dropout rates. This seems to be an issue faced by other researchers in a number of similar studies [25]. In our case, statistical analyses were not possible because of the low number of participants who completed the intervention. In addition, our sample does not accurately represent the broader target population for various reasons. Completers were mainly females. We also failed to recruit participants aged between 15 and 18 years and male participants. This could be because of a lack of expertise in recruitment strategies as outlined above. This suggests that, for future studies, societal outreach as a means of recruitment is necessary. This may include reaching out to contexts where Arabic-speaking youths are present; for instance, schools, municipalities’ different activities for newcomer such as Swedish courses, courses for community information, local communities at suburbs where a high number of immigrants reside, nongovernmental organizations, and other key organizations who have an established relationship with the target population. However, it could also reflect a low acceptability of the treatment or the treatment format. With regard to sampling, there are concerns regarding the occurrence of sampling errors. Some of the noncompleters expressed that the intervention was acceptable. In contrast, many noncompleters expressed not feeling well enough to work on the platform or being under time constraints because of daily responsibilities. Moreover, traces of nonresponse bias were observed.

Younger participants who started the intervention did not complete the intervention. This suggests that there is still room for improvement in terms of developing an acceptable internet intervention targeting participants aged between 15 and 18 years. However, it is worth mentioning that only one participant aged between 15 and 18 years was recruited. During the course of the intervention, some participants expressed that the case studies focused heavily on the journey of refuge (ie, everything that took place from the time they fled their homes to the time of resettlement in the current host country) and that they felt that they currently find themselves in a different phase in life. On the basis of this feedback, culturally adapted interventions may consider focusing more on other aspects of life apart from the journey of refuge. The intended intervention duration of 10 weeks was not followed, and some participants took part in the intervention over a period of 13 weeks. Weekly follow-up calls had to be rescheduled several times because of (1) ill participants, (2) ill therapists because of COVID-19, or (3) participants not being reachable at the scheduled time. This may have affected the engagement of some participants. It should also be mentioned that all the completers were females, indicating that the intervention might be more acceptable for females than for males. This is in line with previous findings from face-to-face psychological interventions, where females tended to adhere to a higher degree than males [68]. To some extent, it also reflects findings of culturally adapted face-to-face treatments for refugee youths [69].

### Conclusions

In conclusion, through this feasibility study, we learned that there is still substantial room for improving the acceptability and feasibility of such an intervention. For example, refinement of recruitment strategies, further simplifying the treatment content, and focusing more on other aspects than the journey of refuge in the cultural adaptation version are all steps in the right direction toward a better fitting intervention for this susceptible group of young individuals. Culturally adapted internet-based intervention studies are scarce and thus, future development and evaluation of such interventions are needed.

## Appendix

Multimedia Appendix 1 Interview guide.

Multimedia Appendix 2 Cultural adaptation.

## Abbreviations

CSQ-3: Client Satisfaction Questionnaire-3
HSCL-25: Hopkins Symptom Checklist-25
iCBT: internet-based cognitive behavioral therapy
ISI: Insomnia Severity Index
PCL-5: Posttraumatic Stress Disorder Checklist
PG-13: Prolonged Grief Disorder-13
PTSD: posttraumatic stress disorder

## Data Availability

The data on which this study was based are not publicly available owing to ethical restrictions; however, the data are available on request from the corresponding author.
