# Supplementary material for: Individually Tailored and Culturally Adapted Internet-Based Cognitive Behavioral Therapy for Arabic-Speaking Youths With Mental Health Problems in Sweden: Qualitative Feasibility Study
Source: JMIR Form Res. 2023 Nov 24;7:e46253. doi: 10.2196/46253 (PMC10709795; doi:10.2196/46253)
Supplement: Multimedia Appendix 1 [file formative_v7i1e46253_app1.docx]

**Interview Guide: Post-treatment***

**General questions (for treatment participants):**

1. [if short on time and when applicable] What was the reason that you discontinued the intervention?
2. In general terms, what can we do to improve the intervention?
3. How did you get information about the study?
4. What led you to sign up/register? (search cause/problem searched for, motivation).
5. Which device did you use to participate in the intervention? (smartphone, computer, tablet)
6. How was your experience reading the material on the homepage? How easy/hard was it to understand?
7. How was your experience answering the questionnaires?
8. How was your experience with the telephone interview?
9. How was your experience logging in to the SahaUng program’s page for the first time?
10. How was your experience with the SMS-authentication?
11. Which language did you choose?
12. Which modules were you allocated?
13. How was your experience of understanding the structure of the webpage and orienting yourself on the SahaUng webpage?
14. How was your experience in understanding the different pathways of communicating with your therapist (in the messaging system and by doing exercises and sending to them)?
15. How should we set up the intervention/treatment to make it more appealing for people with a similar background as yours?

**Questions for treatment participants and non-clinical participants:**

**Acceptability:**

1. What was your overall experience of the modules and the program? – Kindly develop your answer and give examples. AFFECTIVE ATTITUDE
2. How easy or difficult was it for you to understand the texts? - Is there anything that would make the texts easier to understand? INTERVENTION COHERENCE
3. How coherent do you think the modules were? Were the take-home messages comprehensive? INTERVENTION COHERENCE
4. How hard was it for you to read through the modules and comprehend the material provided? BURDEN, OPPORTUNITY COST
5. What obstacles did you experience in completing the intervention and reading the modules and doing the exercises each week? - What would it make it easier? (Planning? Concentration? Confidence in one's own ability? / self-efficacy) OPPORTUNITY COST, BURDEN, SELF-EFFICACY
6. To what extent has your needs been met? PERCEIVED EFFECTIVENESS
7. In a general sense, how satisfied are you with the help you have received? PERCEIVED EFFECTIVENESS
8. Do you have any other feedback you want to share with us? Is there anything that we have not asked about and that you think would be beneficial for us to know? – From your point of view, how can we reach out people with similar background as your own?

**Cultural relevance:**

1. In different countries, cultures and life situations (e.g. being an asylum seeker) there are different ways of looking at and talking about mental illnesses: how well did you feel that the intervention matched your way of looking at and talking about mental illnesses. Did it match the way you usually act to feel better when you are feeling mentally ill? ETHICALITY, CULTURAL RELEVANCE
2. To what extent could you recognize yourself in the cases regarding depression and anxiety?
3. To what extent does the intervention contain relevant linguistic expressions, such as slang?
4. How relevant were the poems and metaphors used in the intervention?
5. To what extent was the material relevant with their regard to your context and life situation?

*This interview guide is a modified version of the supplementary material provided by Lindegaard, Wasteson, Demetry et al. (2022).
