# Supplementary material for: Individually Tailored and Culturally Adapted Internet-Based Cognitive Behavioral Therapy for Arabic-Speaking Youths With Mental Health Problems in Sweden: Qualitative Feasibility Study
Source: JMIR Form Res. 2023 Nov 24;7:e46253. doi: 10.2196/46253 (PMC10709795; doi:10.2196/46253)
Supplement: Multimedia Appendix 2 [file formative_v7i1e46253_app2.docx]

| **Appendix 1*: Cultural adaptation based on Helms (2015)** | | | |
| --- | --- | --- | --- |
| **Module** | **Objectives** | **Type of adaptation** | **Equivalence type** |
| Introduction | - Familiarization with ABC-model. - Goal setting | - Rumi’s poem - Culturally sensitive examples | - Conceptual equivalence - Functional equivalence |
| Depression | - Psychoeducation - Behavioral activation - Acceptance of negative feelings | - Cultural sensitive explanation model (i.e. fleeing from home country 🡪 sadness) - Culturally sensitive examples - Rumi’s poem to “guest house” | - Functional equivalence - Conceptual equivalence |
| Anxiety | - Psychoeducation - Rational using anxiety curve - Exposure technique | - Hafez’s poem - Validation and normalization of having experienced actual danger | - Conceptual equivalence - Functional equvalience |
| Sleep problems | - Psychoeducation - Sleep hygiene strategies such as stimulus control | - Examples related to separation | - Functional equivalence |
| Stress | - Psychoeducation - Balancing obligations and rights | - Examples in light of post-migration stress | - Functional equivalence |
| Worry and rumination | - Psychoeducation - CBT strategies | - Normalization in light of refuge situation | - Functional equivalence |
| Emotion regulation | - Psychoeducation based on DBT - Opposite action | - Culturally sensitive examples | - Functional equivalence |
| Traumatic events | - Psychoeducation - Exposure based on Prolong Exposure therapy | - Relevant cases - Culturally relevant avoidance strategies | - Functional equivalence - Conceptual equivalence |
| Grief and loss | - Strategies based on Shear (2015) | - Intertwining cultural rituals of grieving | - Conceptual equivalence - Functional equivalence - Linguistic equivalence |
| Maintenance | - Sustainability of learnt skills - Management of setbacks | - Culturally sensitive examples | - Functional equivalence |

*This table is a modified version of the supplementary material provided by Lindegaard, Wasteson, Demetry et al. (2022).
